# Editors’ Book Table

**Published:** 1873-07

**Authors:** 


### Editors' Book Table.

[NOTE. — All works reviewed in the columns of the CHICAGO MEDICAL JOURNAL may be found in the extensive stock of W. B. KEEN, COOKE & CO., whose catalogue of Medical Books will be sent to any address upon request.]

BOOKS RECEIVED.

*Illustrations of the Influence of the Mind upon the Body in Health and Disease.* Designed to Elucidate the Action of the Imagination. By DANIEL HACK TUKE, M.D., M.R.C.P., etc., etc.

Mental Physic is but little treated of in the text-books, yet the great majority of the profession know well enough that embarrassment will make a diffident man sweat; that fear will make a coward soon pass a cathartic-like dejection; that "attention and emotional excitement" will bring on uterine pains in an over-sympathetic female friend in attendance in the lying-in-room; that mental anxiety, or suspense, will induce a copious flow of pale urine, or decrease the usual amount of gastric juice. Any one proposing to cure disease, twenty years ago, by using mental means alone, would have been considered of very doubtful veracity. To-day many astonishing cures are accredited solely to mental influences.

The favorite explanation of the curative results of potencies and attenuations, by unbelievers, is that all good is accomplished through the expectation, belief or "imagination" of the patient. Every physician acknowledges that his "success" is almost universally the best in those patients who manifest the most implicit faith in him. On such persons bread pills will act as cathartics, sudorifics, anodynes, or as any other remedy, provided the doctor announces such and such effects are to follow.

Mental influence over the body is an old, old subject. "The mind acts on the body and the body reacts on the mind," is an idea common enough, and it implies two classes of phenomena quite unlike. It is easy enough to persuade ourselves we understand how pathological conditions cause mental disturbance. Abnormal stimuli, malnutrition, imperfect circulation, deformities, and other causes, disturb the brain. But mental influences on the body cannot be so classed. Bodily conditions can be investigated by the microscope, by chemical re-agents, and the various means now taught by physiologists, but mental states cannot be touched, seen, or in any way investigated, except through their effects. Every function of the body has been exhaustively studied, and not a base or an acid in the whole body exists that has not been carefully investigated. Not so thoroughly, however, has the work been prosecuted in mental anatomy and physiology—in fact, this work is only in its infancy. In accounting for effects of pathological conditions on the mind we have something *tangible*, something *known* to work from; but in accounting for effects of mind on body we start from the unknown and possibly unknowable.

Dr. Tuke's idea, in writing this book, is to begin work in this uninvestigated field. It is the first work on this subject extant, and practicing physicians will be amply paid for reading it carefully through, and studying especially the chapter on psycho-therapeutics.

The plan of treating the subject adopted by the author, consists of considering the qualities, relations, and effects on the body of each of the three grand divisions of the mind, viz., the intellect, the emotions, and the will. The influence of each of the three divisions upon sensation, upon the voluntary muscles, (movements, spasms and convulsions, and loss of muscular power,) upon the involuntary muscles, and upon the organic functions, is discussed in separate chapters. Under each division are cited numerous illustrations of great interest. The plan of considering the subject is so well laid out, that every instance of mind influence over the body can be easily classified. By far the most interesting portion of the book is the last chapter but one, on "psycho-therapeutics." Every word of it will be read with absorbing interest, and the only regret one experiences is that no more can be written of a practical character. The character, conduct and ability of the physician at the bedside, are considered highly important elements of success. Hope and confidence are powerful aids to the doctor in effecting curative results, and fear is capable of exercising marvelous therapeutic activity. Exciting the former, and sparingly or rarely employing the latter, are powerful forces for cure in the hands of a perspicacious man. Dr. Rush told us he never prescribed remedies of doubtful efficacy in the critical stage of acute diseases "until he had worked up his patients into a confidence, bordering upon certainty, of their probably good effects." The success of such proceeding oftener answered than disappointed his expectations. Dr. Tuke relates the following:—A very intelligent naval officer suffered violently, for many years, from cramp in the stomach. Anodynes had been exhausted one after another. Bismuth in largest doses had been used and "worn out." On one occasion when suffering severely from the after effects of some form of opium, he was informed that during the next attack he would be put under a medicine, generally believed to be most effective in such cases, although rarely used because of its dangerous qualities—provided he assented. The latter he quickly did. Accordingly, on the first attack after this, a powder containing four grains of powdered biscuit was given every seven minutes, while the gravest concern was manifested in his presence, lest too much be given. The fourth dose caused an entire cessation of pain. Thirty-grain doses of bismuth had never procured relief in less than three hours previously. More powerful than either hope or fear, as a curative agent, is the patient's will. Dr. Laycock asserts that the will can effect such changes in the brain as to arrest an attack of angina or epilepsy. Dr. Tuke quotes the interesting case of Crosse, the electrician, who was bitten by a cat that died the same day of hydrophobia. Three months subsequently he felt, one morning, great pain in his arm, accompanied by extreme thirst. Spasm seized his throat upon attempting to swallow water. It then flashed upon his mind that he was a victim of hydrophobia. His agony of mind was indescribable while contemplating such a death. He concluded he must die. At length he began to reflect, and reflection aroused his will, and he determined he *would not* die. Accordingly he shouldered his gun and went out for the purpose of shooting, his arm aching intolerably the while. "*I walked*," he says, "*the whole afternoon, exerting at every step a strong mental effort against the disease.* When I returned to the house I was decidedly better; I was able to eat some dinner and drink water as usual." In three days the pain had left his arm. He recovered, wholly.

Mesmerism and Braidism are noticed at some length in closing this chapter. No one can determine whether the production of such artificial hypnotism will be used to any great extent to facilitate cures, but that it *is* capable of being so used, every reader of Dr. Tuke's book will concede.

Almost every physician neglects the systematic and scientific employment of physical aids in curing disease, and in so doing one means is thrown away, more powerful than all the agents in the materia medica. This means, empyrics use almost exclusively, and educated medical practitioners ought to divest it of unnecessary accompaniments with which the former envelop it, and incorporate it in the active agents daily employed. J. H. E.

---

